# Antiplatelet Resistance—Does it Exist and How to Measure it?

**Published:** 2009-09-03

**Authors:** S. Saraf, I. Bensalha, D.A. Gorog

**Affiliations:** 1 Cardiology Department, East and North Hertfordshire NHS Trust, UK.; 2 Imperial College, London, UK.

**Keywords:** antiplatelet, resistance

## Abstract

Aspirin and clopidogrel are the most commonly used antiplatelet agents in patients with coronary artery disease. The existence of resistance to these agents has been a controversial issue and new drugs are being developed to overcome this problem. Laboratory tests, which can identify resistance and correlate this with clinical outcome, are being studied in order to identify patients at risk of future thrombotic events. We discuss the evidence for the existence of antiplatelet resistance—both in the laboratory and in the clinical setting. So far, platelet aggregometry has been considered the gold standard test, but is very operator dependant, time consuming, and has shown little correlation with other available tests of antiplatelet resistance. We discuss the available tests of platelet function, their limitations, and evidence for their use. A simple, rapid, near-patient test, which is affordable and useful in the clinical (not just laboratory) setting, could allow risk stratification of patients and individualization of antiplatelet medication to improve outcome.

## Introduction

Platelets are the key players in pathological thrombus formation, that leads to myocardial infarction, ischaemic stroke, and peripheral vascular disease. Aspirin, the oldest antiplatelt agent has shown a significant benefit in the secondary prevention of these ischaemic events. Platelets can be activated through a number of pathways, and antiplatelet agents aim to block one or more of these.

Aspirin and clopidogrel are the antiplatelet agents most commonly used in patients with coronary artery disease. However, some patients continue to experience thrombotic events despite treatment with these agents, and this phenomenon has been termed antiplatelet “resistance”. The exact cause or mechanism that underlies such resistance is unknown; furthermore, the existence of “resistance” has been challenged and remains an issue of much contention.

Nevertheless, it has fuelled the pharmaceutical industry to develop newer drugs, which will be able to “overcome this resistance”. Recent results from the TRITON-TIMI 38^1^ and DISPERSE-2^2^ studies provide promising results for the newcomer antiplatelet agents Prasugrel and AZD6140.

In addition, it has also resulted in a search for a laboratory test to identify patients who exhibit “resistance” to antiplatelet medication, in order to detect those at risk of future thrombotic events.

So far, light transmittance aggregometry has been considered the gold standard test of platelet function. However, this method is highly operator-dependent and has shown little correlation with other available tests of antiplatelet resistance. The ideal test to assess antiplatelet medication should 1) use physiologically relevant agonists to induce platelet activation, 2) be easy to perform (by clinicians), 3) give rapid results within a clinically-relevant timeframe, 4) correlate closely with clinical events, 5) have a high sensitivity and 6) be affordable. None of the available techniques currently fulfils all these criteria.

In this paper, we present the clinical evidence for the existence of antiplatelet resistance, describe the techniques used to date to identify antiplatelet resistance in the laboratory and their relative merits and shortcomings.

## Mechanisms of Action of Antiplatelet Drugs

### Aspirin

The major cyclooxygenase product in platelets is thromboxane A_2_ (TxA_2_) which induces platelet aggregation and acts as a potent vasoconstrictor. Aspirin blocks production of TxA_2_ by acetylation of COX1, the enzyme that produces the cyclic endoperoxide precursor of thromboxane A2. Since platelets do not synthesize new proteins, the action of aspirin on platelet cyclooxygenase is permanent, lasting for the lifetime of the platelet (7–10 days) and repeated doses of aspirin produce a cumulative effect on platelet function. However, aspirin is considered a suboptimal antiplatelet agent since it antagonizes only one particular pathway of platelet activation, leaving several other important pathways unaffected.

### Clopidogrel

Clopidogrel is a thienopyridine derivative. It is a prodrug, oxidized by the hepatic cytochrome P450 system to its active metabolite which irreversibly binds to the ADP-coupled P_2_Y_12_ receptor.^3^ P_2_Y_12_ inhibition thus inhibits ADP-induced platelet activation and resultant aggregation. There is no doubt that clopidogrel is an effective antiplatelet agent, and when added to aspirin, significantly reduces the occurrence of thrombotic events.^4^ Importantly, no direct head-to-head comparisons of aspirin and clopidogrel have been performed in clinical trials. Instead, trials of clopidogrel have assessed its efficacy as an “add-on” therapy to aspirin, presumably to reduce thrombotic events in those patients in whom aspirin may not be totally preventive.

### Ticlopidine

Ticlopidine is another thienopyridine that permanently inhibits the P_2_Y_12_ receptor. It is a prodrug that requires conversion to the active metabolite by the hepatic cytochrome P450 enzyme. It is rapidly absorbed, highly bioavailable and has a prolonged effect. However, its unfavourable side-effect profile with risk of bone marrow suppression has placed it second position with regard to clopidogrel and led to the withdrawal of this drug in some countries (e.g. United Kingdom).

### Prasugrel

Prasugrel is a new oral thienopyridine derivative that produces more potent and irreversible P_2_Y_12_ receptor blockade, with a rapid onset of action. Its active metabolite is R-138727 and it is deemed to be 10 times more potent than currently available thienopyridine derivatives. The JUMBO-TIMI 26^5^ study showed improved platelet inhibition, MACE and reduction in ischemic events with prasugrel compared to clopidogrel. Recent results from the TRITON-TIMI 38^1^ study comparing clopidogrel with prasugrel in 13,608 patients has shown reduced interpatient variability, a 19% relative reduction in the primary endpoint of MACE (p = 0.0004), 24% reduction in myocardial infarction and 52% relative reduction in in-stent restenosis in the prasugrel group. However, bleeding complications were also more frequent with prasugrel, especially intracranial hemorrhage in patients with prior CVA/TIA.

### AZD6140

AZD6140 is an oral and reversible P_2_Y_12_ receptor blocker that does not require hepatic conversion to an active metabolite and produces an overall superior ADP-induced platelet inhibition with less response variability than clopidogrel. It belongs to the cyclopentyltriazolopyrimidine group and has rapid onset and offset of action, which may make it particularly useful in patients who have to undergo imminent surgery. Results from the DISPERSE 2 study showed superior platelet inhibition with AZD6140 when compared to standard dose of clopidogrel in 990 patients with acute coronary syndromes, although this did not translate into a significant reduction in the rates of myocardial infarction.^6^,^7^ The PLATO study will compare AZD6140 with clopidogrel in 18,000 patients with ACS.^2^ AZD6140 is still not licensed for clinical use.

### Cangrelor

This is a potent parenteral P_2_Y_12_ receptor antagonist. It is an ATP analogue with a very rapid onset and a short half-life, with recovery of platelet function in 20–50 minutes after discontinuation of the drug. Its rapid onset of action makes it an attractive option for patients undergoing emergent PCI even if they then need to go on to have bypass surgery. The ongoing CHAMPION study is comparing clopidogrel with cangrelor in patients undergoing PCI.^6^ This drug is still not licensed for clinical use.

### Glycoprotein IIb/IIIa inhibitors

Glycoprotein IIb/IIIa receptors on the platelet surface bind fibrinogen, and are the final common pathway of platelet activation. The GP IIb/IIIa receptor may be activated by any platelet agonist and consequently inhibition of binding to this receptor blocks platelet aggregation induced by any agonist. Three agents approved for use at present are abciximab, eptifibatide and tirofiban. All are effective but need to be given intravenously and are only approved for short-term use.

### Dipyridamole

Dipyridamole interferes with platelet function by increasing the cellular concentration of cyclic AMP. This effect is mediated by inhibition of cyclic nucleotide phosphodiesterase and/or by blockade of available uptake of adenosine, which acts at adenosine A_2_ receptors to stimulate platelet adenylyl cyclase. It has little or no benefit as an antithrombotic drug. In trials in which a regimen of dipyridamole plus aspirin was compared with aspirin alone, dipyridamole provided no additional beneficial effect.^8^ A single study suggests that dipyridamole plus aspirin reduces strokes in patients with prior stroke or transient ischemic attack.^9^

### Cilostazol

Cilostazol is a reversible cAMP phosphodiesterase inhibitor with antiplatelet, antithrombotic and vasodilatory effects. Compared to either placebo or pentoxifylline in six double-blind randomised controlled trials, it has been shown to be effective in reducing intermittent claudication in patients with peripheral arterial disease.^10^

### BM573

BM573 is a thromboxane A2 synthase inhibitor and receptor antagonist. It has been shown to reduce atherosclerosis in LDL-deficient mice, suggesting it may have a role to play in preventing progression of atheroma. It is still in development.^11^

## Laboratory Tests for Monitoring Antiplatelet Therapy

Platelet function tests were devised to detect patients with abnormal platelet reactivity, which may be inborn or acquired. A number of tests are currently available to assess platelet function, some laboratory based and some near patient point of care tests. They have been used in a number of research studies to detect the effect of antiplatelet medication, however none are in routine clinical use as the available tests demonstrate a large variability in the response to antiplatelet medication, with variable prevalence of “resistance”. The most frequently performed platelet function test is platelet or whole blood aggregation induced by ADP or collagen. Platelet aggregometry has been described as the “gold standard” platelet function test, against which other platelet function tests are compared. Flow cytometry is also frequently used to measure platelet activation, and has additional advantages over global tests of platelet function in providing detailed analysis of the surface markers on the platelet. This provides greater insight into the pathomechanism of platelet activation, but may provide less detailed information on platelet activation. Near patient point of care tests are more convenient and provide more readily available test results for clinicians. However, the Scientific and Standardization Committee and the International Society on Thrombosis and Haemostasis do not recommend use of platelet function testing outside research trials, as there is inadequate data addressing the clinical effectiveness of tailoring antiplatelet therapy based on laboratory results of antiplatelet resistance.^12^

### Bleeding time

Dating back as far as 1901, this simple test measures the time it takes for a small skin cut to stop bleeding. It has very poor reproducibility and no study so far has shown it to correlate with bleeding or thrombotic risk.

### Light transmittance aggregometry

Regarded as the gold standard test for assessing platelet reactivity and for validating other, newer tests. Baseline light transmittance is performed on whole blood or platelet fraction, and compared with transmittance following the addition of platelet agonists, such as arachidonate, ADP, thrombin receptor activating peptide, collagen, or epinephrine. Platelets clump in response to these agents and an increase in light transmittance is noted. Subjects whose platelet aggregation is more than 20% with arachidonate are considered aspirin resistant. It is relatively expensive, time consuming, performed on anticoagulated blood and variability in results has been reported.

### Flow cytometry

Here, blood cells are labelled with a fluorescently conjugated monoclonal antibody and are then passed through a flow cytometer, at 1000 to 10 000 cells per minute. They then pass through an active laser light, which activates the fluorophore that is conjugated to the monoclonal antibody. The intensity of fluorescence is directly proportional to the antigen being studied. P selectin (CD62) is expressed on the surface of activated platelets, and helps in formation of the monocyte-platelet aggregates, which are considered to be the most sensitive marker of platelet activation.

### Urinary thromboxane

Urinary thromboxane is a simple test to assess platelet activation through urinary metabolites. Activated platelets synthesize 11-dihydroxy thromboxane B2, an active metabolite of TxA2, and this is detected in urine with an ELISA assay. However, although detection of 11-dihydroxy thromboxane B2 in urine reflects systemic TxA2 formation, 30% is derived from nonplatelet sources and thus falsely high readings may be observed in inflammatory conditions.^13^

### PFA-100

The PFA-100™ System (Dade Behring, Germany) is a semi-automated dual channel device. Blood is drawn into a tube containing 3.2% citrate and allowed to stand for between 30 min and 4 h, after which 800 μl of citrated whole blood is added to each of two preprepared cartridges to wet the filters. Both cartridges contain a membrane coated with type I equine collagen together with an agonist to induce platelet aggregation. In one cartridge the membrane is coated with 10 μM epinephrine and the other with 10 μM ADP. The measurement begins by drawing the blood through a capillary tube and a single aperture (150 μm diameter) into a collagen coated cellulose-acetate filter. This results in the platelets being pre-activated by shear stress of 190 dynes/cm^2^ even before reaching the filters and the agonists. As platelets come into contact with the collagen, they adhere, aggregate and form the primary hemostatic plug, which occludes the aperture (closure time, CT). The greater the platelet inhibition, the longer the closure time.

### Verify now

The Verify Now system (Accumetrics, California) is a platelet function assay utilizing light source to detect the amount of platelet aggregation. Platelets adhere to the fibrinogen coated beads in the tube, aggregate and fall out of solution, changing the extent and rate of light transmittance. Light transmittance is inversely related to the amount of platelet aggregation. There are 3 types of assays available. The Aspirin assay utilizes arachidonic acid as an agonist to assess the antiplatelet effect of aspirin. Similarly, the P2Y12 assay utilizes ADP as an agonist to assess the effect of clopidogrel and the IIbIIIa assay utilizes a thrombin receptor activating peptide as an agonist to assess the response to IIbIIIa inhibitors.

### TEG 5000 thromboelastograph haemostasis system

Thrombelastography (Haemoscope, USA) measures all phases of haemostasis from clot formation to clot lysis. Blood is held in a cylindrical cup which oscillates through an angle of approximately 5 degrees. A pin is suspended into this blood by a torsion wire, and monitored for motion. The strength of the fibrin platelet bond during clot formation affects the magnitude of the pin motion, giving an idea of overall haemostasis. TEG Platelet Mapping technology allows estimation of the percentage inhibition of platelet aggregation by aspirin, clopidogrel or GPIIb/IIIa inhibitors, thus allowing tailoring of individual antiplatelet therapy.

### Global Thrombosis Test (GTT)

The Global Thrombosis Test (GTT) (Montrose Diagnostics Ltd, UK) is a novel platelet function test, which is currently the most physiological test of platelet reactivity, in that the technique is performed on non-anticoagulated, native blood, without added external agonists. In this technique, an occlusive thrombus is formed using high shear stress, analogous to that in a stenosed coronary artery. This first phase of the test creates an occlusive thrombus under conditions of high shear and is used as marker of platelet function, the more reactive the platelets, the faster the occlusion will occur (occlusion time, seconds). The restart of blood flow following occlusion is due to spontaneous thrombolysis (lysis time, seconds). This is a near-patient test, which provides a result within 10 minutes on the patient’s thrombotic status, and is thus highly applicable to acute clinical situations, as well as more general screening. Studies correlating GTT with clinical outcomes are currently in progress and early results suggest it may have an important role in clinical practice.^14^

## Limitations of Platelet Function Tests

Most tests lack sensitivity, have low positive predictive value for clinical events and are difficult to perform in the clinical setting. Furthermore, most tests are performed on anticoagulated blood or use supra-high doses of agonists to induce platelet aggregation, so the physiological relevance of such tests remains questionable. There is clearly a need for a truly physiological, reliable, reproducible and clinically relevant test.^15^ Tests such as aggregometry and flow-cytometry are time consuming, need special expertise to perform and are not applicable to providing a rapid result in the clinical setting to influence practice. A common drawback with many platelet function tests has been the lack of serial measurements. Furthermore, most studies have been carried out on a small number of patients and there has been no published study suggesting an improvement in clinical outcome with tailoring of antiplatelet medication based on the results of platelet function tests. Recent studies comparing the results with different platelet function tests noted there was wide variability and poor correlation amongst them.^15^,^16^

## Evidence that Antiplatelet Resistance Exists

### Definition of “resistance”

The term “resistance” as applied to antiplatelet medication implies an endogenous mechanism in certain individuals, which prevents the drug from exerting its full antithrombotic effects. This is however, a misnomer. Almost all drugs known to man exert varying effects in different individuals and this should not be termed resistance. The causes for this are plentiful, and listed below. The proposed mechanisms underlying antiplatelet resistance to aspirin and clopidogrel are summarised in Table 1.

From a clinical point of view, the term “resistance” has been used to describe the ongoing thrombotic events that occur in some individuals despite taking antiplatelet medication. However, the laboratory phenomenon of resistance is based on the results of platelet function tests, which show incomplete inhibition of aggregation by the medication in question. We will now discuss the evidence for existence of “antiplatelet resistance”.

## Laboratory Evidence of Antiplatelet Resistance

The prevalence of aspirin “resistance” is considered to be between 5%–60%, with a similar prevalence of clopidogrel resistance. Studies reporting aspirin resistance are summarized in Table 2. Studies reporting on the phenomenon of clopidogrel resistance are summarized in Table 3. The prevalence of antiplatelet resistance varies with the laboratory method used, the drug studied, the drug dose and with the disease state. As shown in Tables 1 and 2, there is wide variation in the reported prevalence of antiplatelet resistance and lack of consistency between studies.

## Prevalence of Antiplatelet Resistance in Different Populations^17^,^18^

### Stable coronary artery disease

A prospective study of 326 patients with stable CAD revealed the incidence of aspirin resistance was 5.5% when assessed by LTA and 9.5% when assessed using the PFA-100.^19^,^20^ Another study of 98 subjects showed 30% resistance in patients on aspirin 160 mg/d using the PFA-100,^21^ with another study using the PFA-100 reporting a 27% incidence.^22^

### Acute coronary syndrome

Among 104 ACS patients tested with the PFA-100, the incidence of aspirin resistance was found to be 40%.^22^ The Warfarin Aspirin Reinfarction II Study (WARIS-II) study allocated 202 patients to receive aspirin, warfarin or both. Aspirin resistance was observed in 35% of subjects taking aspirin alone and in 40% taking aspirin and warfarin. Major adverse cardiac events occurred more frequently in aspirin non-responders compared to responders (36% vs. 24%, p = 0.28).^23^

### Elective PCI

The VerifyNow assay was used to detect aspirin resistance in 151 Asian patients undergoing elective PCI. All patients had been taking 80–325 mg aspirin for at least a week prior to the procedure, yet 19% were found to be aspirin resistant.^24^

### In-stent restenosis

In a study of 204 patients, 31% patients with in-stent restenosis were found to be aspirin resistant using PFA-100, compared to 11% of those with patent stents (p < 0.001).^25^

## Clinical Significance of Antiplatelet Resistance

The concept of antiplatelet resistance is variably defined. It is not clear whether the definition of antiplatelet resistance should be based on laboratory results or clinical outcomes. However, several recent studies suggest that antiplatelet therapy resistance is associated with an increase in the risk of adverse cardiovascular outcomes in patients with CAD, CVA or PVD (Table 4 and Table 5).

### Stable CAD

A study by Gum and co-workers (using optical platelet aggregation) evaluated 326 patients with stable CAD receiving aspirin for greater than 7 days. Aspirin-resistance was identified if the aggregation was greater than 70% in response to 10 micromolar ADP or greater than 20% with 0.5 mg/mL of arachidonic acid. Based on this definition, 5.5% of patients were “resistant” and had a significant increase in the combined endpoint of death, MI or stroke over a follow up period of nearly 2 years, compared to responders.^26^

### Elective PCI

The VerifyNow assay was used to detect antiplatelet resistance to GPIIb/IIIa inhibitors in 485 patients undergoing elective PCI. Patients whose platelet function was inhibited by 90% or more had an event rate of 2% compared with 10% for patients with inhibition of less than 90%.^27^

Holzholter measured platelet reactivity in 802 patients undergoing elective PCI after 600 mg clopidogrel loading and concluded that patients with high platelet reactivity had a worse clinical outcome at 30 days.^28^

### Primary angioplasty

Reduction in platelet aggregation in response to high dose loading treatment with aspirin and clopidogrel was assessed in 60 patients presenting with acute ST elevation MI undergoing primary PCI. They were divided into 4 quartiles based on reduction in platelet aggregation using the cone and plate aggregometer. Patients in the first quartile had platelet aggregation 103% ± 8%, whereas those in the 2nd, 3rd and 4th quartile had platelet aggregation of 69, 58 and 33% of their respective baselines. At 6 months’ follow-up, 7 patients in the first quartile and 1 patient in the 2nd quartile had experienced a cardiovascular event.^29^

### Stent thrombosis

Gurbel and coworkers compared 20 patients with subacute stent thrombosis (SAT) to 100 patients undergoing PCI who did not experience SAT. Using LTA to assess platelet reactivity and VASP to assess clopidogrel effect, the results suggested that high post treatment platelet reactivity and incomplete inhibition of P_2_Y_12_ are risk factors for stent thrombosis.^30^ In another study of 105 patients undergoing elective PCI, 5%–11% were identified as being clopidogrel non-responders and among these patients, 2 SATs were documented.^31^

In a well-conducted large, prospective study, Bounamici and co-workers measured platelet aggregation in response to ADP in 804 patients undergoing coronary intervention with a drug eluting stent and followed them up for 6 months. The incidence of SAT was 8.6% in nonresponders (105 patients) and 2.3% in responders suggesting a strong correlation between stent thrombosis and clopidogrel resistance.^32^ Another small study showed that 10 patients with SAT had significantly greater shear-induced platelet aggregation compared to PCI patients who had not experienced SAT or compared to normal controls, indicating that resistance to antiplatelet therapy and increased shear induced platelet aggregation correlated well with stent thrombosis.^33^

The interval between SAT and assessment of antiplatelet resistance in all the above studies was variable. Also, the patient population was diverse hence further trials are required to assess the relation between stent thrombosis and antiplatelet resistance mechanisms.

### Cerebrovascular disease

A small study compared 35 patients with symptoms (ischemic stroke or TIA) in the preceding 3 days to 18 patients without symptoms (no CVA symptom for >24 months), all of who had been taking aspirin for at least 5 months. Using the PFA-100, 34% of symptomatic patients were identified as aspirin resistant compared to none of the asymptomatic patients.^34^

Among 180 stroke patients, aspirin resistance was identified in 33%. All patients were followed up for 2 years and major end points were observed in 40% of aspirin resistant patients compared with 4% of aspirin responders (p < 0.0001).^35^

### Diabetes

Angiolillo and colleagues assessed platelet reactivity using LTA and flow cytometry in 173 type 2 diabetics with CAD on dual antiplatelet therapy, and showed that patients with high platelet reactivity (HPR) had an increased risk of MACE with a hazard ratio of 3.35. High platelet aggregation in diabetic patients may be secondary to various factors such as decreased nitric oxide production, increased sensitivity to ADP and overproduction of leptin receptors secondary to obesity.^36^ The OPTIMUS study showed HPR in 60% of diabetic patients despite treatment with 150 mg daily clopidogrel, suggesting that high doses of clopidogrel may not overcome the increased platelet reactivity in certain population subgroups.^37^

### Peripheral vascular disease

A study of 100 patients with intermittent claudication undergoing elective ileofemoral balloon angioplasty assessed for aspirin resistance using whole blood aggregometry at baseline and at regular intervals for up to a year post angioplasty. Reocclusion at the site of angioplasty during follow up occurred exclusively in patients who had been identified as being aspirin resistant.^38^

## Management of Antiplatelet Resistance

The clinician is currently able to partially improve the responsiveness to antiplatelet therapy by acting on extrinsic factors, involved in the aetiology of resistance, including compliance to treatment, drug-drug interactions and good control of blood pressure, glycaemia and lipid levels. Several studies have shown that clopidogrel loading with 600 mg has a stronger and faster inhibitory effect on platelet reactivity than the 300 mg loading dose.^39^,^40^ Increasing the loading dose to 900 mg has not been shown to be of benefit, indicating a threshold to the platelet inhibitory effect of clopidogrel.^41^,^42^

The CLEAR-PLATELETS study showed that Clopidogrel loading combined with eptifibatide resulted in reduced myocardial necrosis compared to standard or high loading dose of clopidogrel alone.^43^

The ISAR-CHOICE-2 study demonstrated the beneficial effect on platelet inhibition of increasing the maintenance dose of clopidogrel to 150 mg.^44^

In the ARMYDA-4 study, reloading with 600 mg clopidogel pre PCI did not confer any additional benefit in patients on chronic clopidogrel therapy. The ARMYDA-5 study, which compared Clopidogrel loading with 600 mg “in lab” vs. 4–8 hours pre PCI, did not show any significant difference in outcome in the two groups, but this study was underpowered to detect a significant difference.^45^,^46^

Results from the recent ARMYDA-PRO study suggest high pre PCI platelet reactivity using the VerifyNow P2Y12 assay may predict MACE at 30 days.^47^ Use of point of care platelet function tests may help in identification of these high-risk patients, and assist the clinician in optimising their antiplatelet medications.

However, there still remains controversy over the benefit and the safety of high loading and maintenance doses of clopidogrel.

Several studies are focusing on this issue, including the CURRENT/OASIS-7 trial. This large population-based study will evaluate whether high dose clopidogrel and/or aspirin improves clinical outcome or increases bleeding risk.

Alternative antiplatelet drugs, including novel P_2_Y_12_ ADP receptor antagonists are currently under clinical investigations. Results from the recent TRITON-TIMI 38 study show that prasugrel significantly reduced the rates of recurrent ischemic events, including stent thrombosis, although this was offset by an increase in major bleeding.^1^ The GRAVITAS study is currently underway to assess whether tailoring the dose of antiplatelet medication based on the results of the VerifyNow assay improves clinical outcomes.

Whether higher doses of aspirin and/or clopidogrel are sufficient to overcome the “resistance” seen in some individuals on low doses of these drugs, is not known. How higher doses of aspirin and/or clopidogrel compare to novel antiplatelet drugs with respect to their antiplatelet effects and specifically, in patients who are “resistant” to low dose aspirin/clopidogrel is again, unknown. The relative bleeding risks with these regimens has also not been evaluated.

## Conclusion

There is no doubt that the laboratory phenomenon of “resistance” to antiplatelet medication exists. There are many tests to assess platelet reactivity and these have demonstrated a large variability in the response to antiplatelet medication, with variable prevalence of “resistance”. The definition of “resistance” is fraught with difficulty as the different methods report different prevalences, depending on the test used, the cut-off value used to define resistance, the timing with respect to medication and the population studied. A metanalysis by Hovens et al found heterogeneity in the prevalence of aspirin resistance, and this was due to the variability in results using different platelet function tests.^48^

It is extremely difficult for clinicians to determine which method to use to assess platelet function and how to interpret the results. There has been no good correlation so far amongst the various different platelet function tests. Many are time consuming, and not applicable to a clinical setting. None fulfils the “ideal” criteria described in our introduction. Furthermore, to date, there is very little data to suggest that altering antiplatelet medication based on the results of laboratory tests of “resistance” improves clinical outcomes.

A metanalysis by Snoep et al^49^ suggests patients with laboratory aspirin resistance are more likely to experience adverse cardiac events, but it is important to point out that no prospective, well-powered clinical trial has assessed the benefit of tailoring antiplatelet medication specifically to populations with increased platelet reactivity. This is partly because we do not know which test or tests best define antiplatelet resistance and which medication(s) best improve outcome in these patient populations. The approach to the problem of antiplatelet resistance has been to develop newer drugs to further inhibit platelet reactivity or increase the dose and timing of treatment with currently available antiplatelet agents. However, both these approaches have been targeted at “allcomers”, rather than specifically tailoring either of these approaches to those patients identified as being non-responders. Importantly, the common side-effect of bleeding with all antiplatelet medications means that the risk vs. benefit ratio needs to be carefully balanced, and it may be more important to individualize such medication to subjects identified as “resistant” rather than giving stronger medication or higher doses to allcomers. Furthermore, the prevalence of “resistance” to these newly developed antiplatelet medications has not been evaluated.

We believe a simple, rapid, near-patient test, which is affordable and useful in the clinical (not just laboratory) setting needs to be validated in a large scale clinical trial, to identify patients with impaired response to antiplatelet medication. This would allow risk stratification and individualization of antiplatelet medication to improve outcome in these patients, with novel treatments or optimised doses of currently available drugs.

## Figures and Tables

**Table 1.** Proposed mechanisms underlying antiplatelet resistance.

Reduced Bioavailability	Non compliance
	Inadequate dose
	Poor absorption (e.g. enteric coated aspirin)
	Increased metabolism
	Interaction with drugs involving the cytochrome P-450 CYP3A4 system (important for prodrugs converted by this system to the active metabolite, e.g. clopidogrel)
	Drug interaction-e.g. NSAIDS, ACE inhibitors^65^
Genetic variation	Mutation of COX1 gene
	Poymorphisms in the COX1, GPIa/IIa, GP IIIa, P_2_Y_1_ or P_2_Y_12_ genes
	Polymorphism P-450 CYP3A gene (for clopidogrel and other prodrugs metabolised by this system)
	Platelet glycoprotein receptor polymorhism
	Overexpression of COX2 in platelets and endothelial cells
Enhanced platelet turnover	Increased platelet production by the bone marrow
	Introducing new platelets not exposed to aspirin or clopidogrel (e.g. transfusion)
	Cigarette smoking induced platelet activation^66^–^68^
	Increased erythrocyte induced platelet activation^69^–^72^
Alternate pathways of platelet activation	TXA2 synthesis induced by cytokines, by oxidative stress or by nucleated cells
	Catecholamine induced platelet activation due to excessive exercise and mental stress^73^–^76^
	High shear stress, collagen, thrombin and other pathways of platelet activation
Individual variation	Diabetes or Insulin Resistance
	Hypercholesterolemia
	Hypertension
	Elderly patients
	Obesity
	Sexual variation

**Table 2.** Summary of laboratory tests reporting Aspirin resistance.

**Study**	**n**	**Type of subjects**	**Aspirin dose**	**Platelet function test**	**Prevalence of resistance (%)**
Gum et al^50^	325	Stable CAD	325 mg	ADP and AA induced optical aggregation	5.2
Mueller et al^38^	100	PAD	100 mg	Corrected whole blood aggregometry	60
Grotemeyer et al^35^	180	CVA	1500 mg	Platelet reactivity	33
Chen et al^24^	151	Elective PCI	80–325 mg	RPFA	19
Andersen et al^23^	202	Post MI	160 mg Aspirin vs. 75 mg Apirin plus warfarin	PFA-100	35% in patients taking aspirin only, vs. 40% in patients taking aspirin and warfarin
Macchi et al^21^	98	Stable CAD	160 mg	PFA-100	29%
Helgason et al^51^	306	CVA	300–325 mg	ADP induced platelet aggregation	25%
Hobikoglu et al^22^	204	ACS: 104 Stable CAD: 100	80–300 mg	PFA-100	40% in ACS 27% in Stable CAD
Grundmann et al^52^	53	CVA/TIA in prev 3 days 35	100 mg	PFA-100	34% in symptomatic 0% in asymptomatic patients
Alberts et al^53^	129	CVA	81 mg vs. 325 mg	PFA-100	37% overall, with 56% in patients on 81 mg vs. 28% in those on 325 mg aspirin.

**Table 3.** Summary of laboratory tests reporting clopidogrel resistance.

**Study**	**n**	**Condition studied**	**Loading dose clopidogrel**	**Maintenance dose clopidogrel**	**Platelet function test**	**Prevalence of resistance**
Gurbel et al^54^	92	PCI	300 mg	75 mg	LTA	31%–35%
Angiolillo et al^55^	52	Diabetes	300 mg	75 mg	LTA and Flow cytometry	38% in DM, 8% in non-DM
Angiolillo et al^56^	48	PCI	300 mg	75 mg	LTA	44%
Lepantalo et al^57^	50	PCI	300 mg	75 mg	LTA and PFA 100	40%
Jaremo et al^58^	18	PCI	300 mg	75 mg	LTA	28%
Lev El et al^59^	150	PCI	300 mg	–	LTA	24%
Mobely et al^60^	50	PCI	300 mg	75 mg	LTA	30%
Muller et al^61^	115	PCI	600 mg	75 mg	LTA	5%–11%
Barragan et al^62^	48	ISR^16^ vs no ISR^32^		Clop 75 mg B.I.D. vs. Ticlopidine 250 mg B.I.D.	Flow cytometry	63% (ISR) vs. 40% (no ISR)
Bounamici et al^32^	804	ISR	600 mg	75 mg	ADP induced platelet aggregation	13%
Ajzenberg et al^63^	32	ISR^10^ vs. no ISR^22^	300 mg	75 mg	Shear induced platelet aggregation (SIPA)	41% (cases) vs. 18% (controls) at shear rate of 200/s 57% (cases) vs. 23% (controls) at shear rate of 4000/s
Matetzky et al^29^	60	STEMI	300 mg	75 mg	LTA	25%
Dziewierz et al^64^	31	CAD	300 mg	–	LTA	23%

**Table 4.** Summary of recent studies reporting on the clinical correlates of laboratory antiplatelet resistance to aspirin.

**Study**	**Population**	**Follow-up**	**Method**	**Primary endpoint**	**Clinical implications**
Gum et al^26^	Stable CAD (n = 326)	2 yrs	Optical aggregometry	MACE	5.2% resistance, associated with increased risk (hazard ratio 3.12) of CV death, MI or stroke
Substudy of HOPE^77^	Patients with MI, stroke or CV death (n = 488)	5 y	Urinary thromboxane metabolite levels	MACE	Patients in the upper quartile had 1.8 times higher risk than those in the lower quartile (p = 0.009)
Mueller et al^78^	Intermittent claudication undergoing peripheral angioplasty (n = 100)	18 m	Corrected whole blood aggregometry	ISR	Risk of reocclusion at the site of angioplasty was 87% higher in patients with failed inhibition of aggregation to collagen and ADP
Pamukcu et al^79^	ACS (n = 105)	1 y	PFA-100	MACE	MACE occurred in 45% of patients with aspirin resistance and in 12% in aspirin-sensitive patients
Grotemeyer et al^80^	Cerebrovascular disease (n = 174)	2 y	Platelet reactivity test- Residual number of platelets in supernatant of centrifuged samples^81^	MACE	Recurrent stroke, MI or vascular death was more likely to occur in aspirin non-responders compared with responders (40 vs. 4.4%, p < 0.001)

**Table 5.** Summary of recent studies reporting on the clinical correlates of laboratory antiplatelet resistance to clopidogrel.

**Study**	**Population**	**Follow-up**	**Method**	**Endpoint**	**Clinical relevance**
Matetzky et al^82^	STEMI undergoing primary PCI (n = 60)	6 months	ADP induced aggregation using LTA	MACE	Patients with recurrent cardiac events had lower percentage reduction of ADP-induced platelet aggregation 91 ± 21 vs. 62% ± 21% percent of baseline, p < 0.001 (day 3) and 90 ± 16 vs. 64% ± 27%, p < 0.001 (day 6)
Barragan et al^83^	SAT (n = 16) vs. No SAT (n = 30)	Retrospective	Enhanced platelet reactivity using VASP assay	NA	Significant difference in VASP assay between SAT (63.28% ± 9.56%) vs. no SAT (39.80% ± 10.9%) P < 0.0001
Ajzenberg et al^84^	N =49 SAT = 10 (cases) NoSAT = 22 (Controls) Healthy = 17	Retrospective	Shear induced platelet aggregation (SIPA)	NA	SIPA higher in cases than in controls 41 ± 12 vs. 18 ± 8%, p = 0.013 at shear rate of 200/s and 57 ± 16 vs 23% ± 21%, p = 0.009 at shear rate of 400/s
CREST^85^	N = 120 SAT = 20 NO SAT = 100	Retrospective	High post-treatment reactivity assessed by LTA and incomplete P_2_Y_12_ receptor inhibition assessed by VASP	NA	Increased platelet reactivity in patients with SAT 49 ± 4 vs. 33% ± 2% for 5 μmol/l ADP-induced aggregation, p < 0.05 and 65 ± 3 vs. 51% ± 2% for 20 μmol/l ADP-induced aggregation, p < 0.001
Cuisset et al^86^	106 ACS patients undergoing PCI	1 month	LTA assessed at the time of the intervention	MACE	Patients with recurrent CV events had a significantly higher ADP-induced platelet aggregation (p < 0.0001)
EXCELSIOR^87^	802 undergoing elective PCI pre-treated with 600 mg loading dose clopidogrel	1 month	ADP-induced platelet aggregation assessed by LTA	MACE	MACE increased with quartiles of ADP-induced platelet aggregation, i.e. 0.5% in the 2 quartiles with the lowest platelet aggregation 3.1% in the third quartile 3.5% in the highest quartile (p = 0.034).
Gurbel et al (PREPARE POST-STENTING)^88^	192 patients undergoing non emergent PCI 36-UA 11-ACS 145-Stable IHD	6 months	ADP induced platelet aggregation assessed by LTA	MACE	Post treatment ADP-induced aggregation by LTA was greater in those patients with recurrent events compared to event free patients (63 ± 12 vs. 56% ± 15%, p = 0.02)
Lev et al^89^	150 patients undergoing elective PCI	–	LTA	–	The percentage of patients with high post-clopidogrel ADP-induced aggregation (>75th percentile) was higher among aspirin-resistant than aspirin-sensitive patients (5 μmol/l ADP: 79 vs. 18%, p = 0.001; 20 μmol/l ADP: 73 vs. 19%, p = 0.001).
Geisler et al^90^	379 PCI patients (206 stable CAD and 173 with ACS) treated with 600 mg clopidogrel loading	3 months	Assessment of response to clopidogrel using LTA	MACE	MACE more frequent in clopidogrel non-responders than in those sesnsitive to clopidogrel (23 vs. 6%; p = 0.004).

## Abbreviations

ACS: acute coronary syndrome;
ADP: adenosine diphosphate;
CAD: coronary artery disease;
CVA: cerebrovascular accident;
DM: diabetes mellitus;
GP: glycoprotein;
IHD: ischaemic heart disease;
ISR: in-stent restenosis;
LTA: light transmission aggregometry;
MACE: major adverse cardiac events;
MI: myocardial infarction;
PCI: percutaneous coronary intervention;
PFA-100: platelet function analyser 100;
PVD: peripheral vascular disease;
SAT: subacute stent thrombosis;
STEMI: ST elevation myocardial infarction;
TRAP: thrombin receptor activating peptide
